# Supplementary material for: Tead1a Initiates Transcriptional Priming Through the TEAD1a/YAP‐Notch1‐Spi1/Cebpα Axis to Promote Neutrophil Fate
Source: Adv Sci (Weinh). 2025 Aug 28;12(41):e05441. doi: 10.1002/advs.202505441 (PMC12591127; doi:10.1002/advs.202505441)
Supplement: Supplementary file 4 — Supplementary Dataset [file ADVS-12-e05441-s004.zip › 3-3.zip!/3-3/02-3.cluster/cluster6/Enrich/ego_MF.pdf]

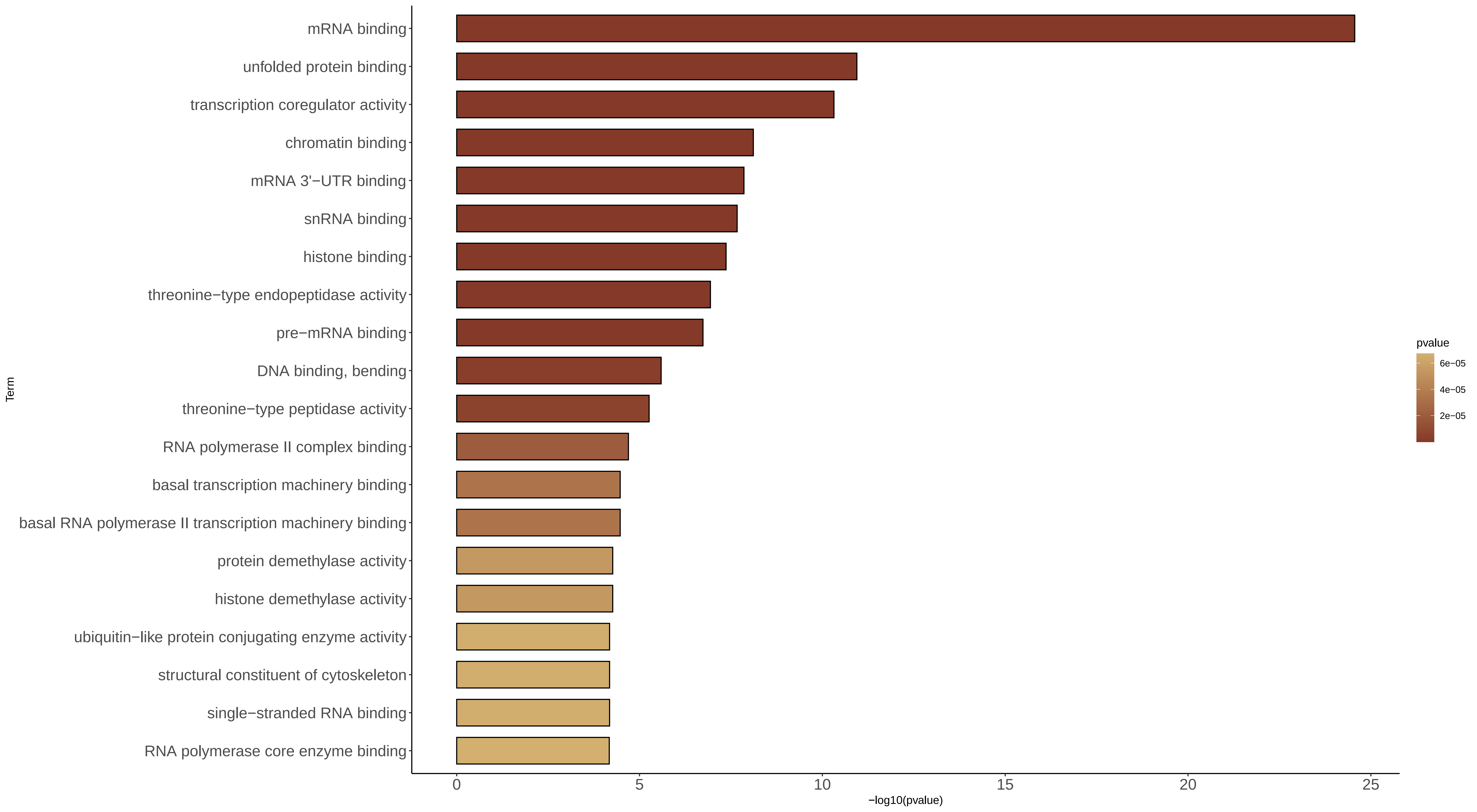
mRNA binding
unfolded protein binding
transcription coregulator activity
chromatin binding
mRNA 3'−UTR binding
snRNA binding
histone binding
threonine−type endopeptidase activity
pre−mRNA binding
DNA binding, bending
threonine−type peptidase activity
RNA polymerase II complex binding
basal transcription machinery binding
basal RNA polymerase II transcription machinery binding
protein demethylase activity
histone demethylase activity
ubiquitin−like protein conjugating enzyme activity
structural constituent of cytoskeleton
single−stranded RNA binding
RNA polymerase core enzyme binding
Term
0
5
10
15
20
25
−log10(pvalue)
pvalue
6e−05
4e−05
2e−05
